# Evidence for intron length conservation in a set of mammalian genes associated with embryonic development

**DOI:** 10.1186/1471-2105-12-S9-S16

**Published:** 2011-10-05

**Authors:** Cathal Seoighe, Paul K Korir

**Affiliations:** 1 National University of Ireland, Galway, University Road, Galway, Republic of Ireland

## Abstract

**Background:**

We carried out an analysis of intron length conservation across a diverse group of nineteen mammalian species. Motivated by recent research suggesting a role for time delays associated with intron transcription in gene expression oscillations required for early embryonic patterning, we searched for examples of genes that showed the most extreme conservation of total intron content in mammals.

**Results:**

Gene sets annotated as being involved in pattern specification in the early embryo or containing the homeobox DNA-binding domain, were significantly enriched among genes with highly conserved intron content. We used ancestral sequences reconstructed with probabilistic models that account for insertion and deletion mutations to distinguish insertion and deletion events on lineages leading to human and mouse from their last common ancestor. Using a randomization procedure, we show that genes containing the homeobox domain show less change in intron content than expected, given the number of insertion and deletion events within their introns.

**Conclusions:**

Our results suggest selection for gene expression precision or the existence of additional development-associated genes for which transcriptional delay is functionally significant.

## Introduction

One of the salient features of eukaryotic genomes is the pervasive presence of introns, comprising up to 95% of transcribed primary protein-coding sequences in mammals [1]. The functions, origins and evolutionary trajectory of introns have long been of great interest in genomics and genome evolution. Although some theories of the spread of introns postulate that they can accumulate passively as a consequence of insufficient purifying selection to remove them in organisms with relatively low effective population sizes [2,3], introns have been shown to play a number of functional roles [4]. They give rise to the possibility of alternative splicing, contributing to the diversity of biomolecules, and they can also have a substantial impact on levels of gene expression [5-7]. Introns contain most of the sequence features required for splicing (5’ and 3’ splice sites, branch point sites (BPS), poly-pyrimidine tracts and various intronic splicing elements). Many introns also contain functional non-coding RNAs [4], which can play critical roles in fine-tuning gene expression [8]. Lastly, introns have been proposed to control the timing of gene expression by delaying transcription [9].

Negative feedback loops with a time delay can result in oscillating patterns of gene expression, which can be exploited by living organisms as biological time-keeping devices. This appears to be particularly important in development [9]. The hairy and enhancer of split 7 (*Hes7*) gene is involved in the control of somite formation through oscillatory patterns of gene expression [10]. Recently, Takashima *et al.*[*11*] investigated *in vivo* the impact of removing the introns from the mouse *Hes7* gene. They found that expression of the mutant *Hes7* gene occurred approximately 19 minutes earlier than for the wild-type and that this reduction in gene expression delay resulted in abolition of oscillations and segmentation defects. Given that the delay associated with transcribing introns appears to play a crucial role in the functioning of this gene, the length of the introns may be evolving under purifying selective pressure. Moreover, further examples of genes with highly conserved intron content across species may reveal more genes for which transcriptional delay is an important aspect of gene regulation. We investigated the conservation of the intron content of *Hes7* across a diverse set of nineteen mammals and carried out a search for other genes for which total intron length shows evidence of evolutionary conservation. In such cases introns are likely to play an important functional role and, in a subset, time delays associated with transcribing introns may be a significant aspect of gene regulation.

## Results and discussion

**Figure 1:**
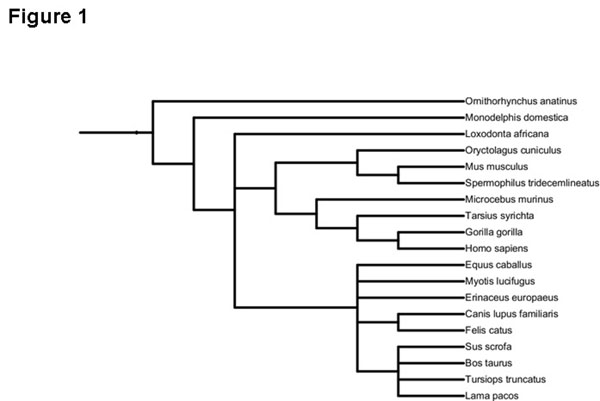
**Phylogeny** Phylogenetic tree illustrating relationships of the 19 mammals included in this study.

Delayed expression of *Hes7* resulting, at least in part, from intron transcription has been proposed to play a key role in somite formation in animal embryos by establishing an oscillating pattern of gene expression [11-14]. Given the important role played by the length of the introns rather than their specific sequence content in this gene, we compared the combined length of *Hes7* introns across a diverse set of mammalian species (Fig. 1) to determine the extent to which the intron content of this gene is conserved over evolutionary time. For each orthologue, the sum of the distances between successive exons in the canonical transcript (according to Ensembl) was calculated. Despite the fact that there may be differences in gene annotation across species the intron content of most of the orthologues was similar. Nine out of the twelve of the available orthologues of *Hes7* among the mammalian species in our dataset differed in combined intron length from the human gene by less than 10% (Table 1).

**Table 1:** **Conservation of Hes7 intronlength** Intron content of orthologues of human *Hes7* (OrthoDB ID EOG45TDC4). The tarsier orthologue was omitted. This gene had no annotated introns but the annotation appeared to be incomplete.

Species	Common name	Gene	#introns	CIL^1^
*Homo sapiens*	Human	ENSG00000179111	3	1839
*Mus musculus*	Mouse	ENSMUSG00000023781	3	1846
*Oryctolagus cuniculus*	Rabbit	ENSOCUG00000002606	3	1696
*Canis familiaris*	Dog	ENSCAFG00000016957	3	1823
*Felis catus*	Cat	ENSFCAG00000015190	3	1958
*Equus caballus*	Horse	ENSECAG00000013278	3	1804
*Myotis lucifugus*	Little brown bat	ENSMLUG00000008014	3	1810
*Erinaceus europaeus*	Hedgehog	ENSEEUG00000006336	3	2774
*Sus scrofa*	Pig	ENSSSCG00000017982	4	1550
*Tursiops truncatus*	Bottlenose dolphin	ENSTTRG00000010312	3	1827
*Loxodonta africana*	African elephant	ENSLAFG00000002331	3	1870
*Monodelphis domestica*	Grey short-tailed opossum	ENSMODG00000007704	3	2364
*Ornithorhynchus anatinus*	Platypus	ENSOANG00000022456	2	2018
*Bos taurus*	Cow	ENSBTAG00000012436	3	2364
*Gorilla gorilla*	Gorilla	ENSGGOG00000005760	3	1840

^1^Cumulative intron **length**

To determine whether intron content, and thus potentially transcription time, of *Hes7* was exceptionally conserved compared to other genes and to discover additional examples of genes for which there is evidence of intron content conservation we compared intron content (defined as the sum of the lengths of all introns in the canonical transcript) between human gene models from Ensembl and sets of orthologous genes, obtained from OrthoDB [15]. Because the pattern of insertion and deletion may differ between very large and smaller introns we restricted to genes with introns of similar lengths to *Hes7* (specifically we considered genes for which the sum of the intron lengths in the canonical transcript was between one and five kilobases). Restricting also to orthologous groups represented in at least half of the species, only 11 other orthologous groups (from a total of 1875 satisfying these criteria) had intron content within 10% of the human gene in as high a proportion of the orthologues as the *Hes7* group. Remarkably, of the corresponding 12 human genes (including *Hes7*), five are annotated with the gene ontology (GO) term GO:0007389 (pattern specification process) from the biological process component of GO, defined as “any developmental process that results in the creation of defined areas or spaces within an organism to which cells respond and eventually are instructed to differentiate”. Using the DAVID functional annotation tool [16,17], this is the most statistically enriched GO term (p = 9.4 × 10^–5^) and remains significant following correction for multiplicity of testing using the Benjamini-Hochberg method (FDR = 0.03). Six of the genes were annotated with the Swissprot and Protein Information Resource (SP-PIR) keywords term ‘developmental protein’ (FDR = 0.003).

We also used a more relaxed conservation threshold and identified a larger set of 144 genes with relatively conserved intron content among the genes with between one and five kilobases of intron. Genes for which more than 50% of the orthologues have intron content within 10% of the intron content of the human member of the group were included in this group, again restricting to orthologue groups represented in at least half of the species. We again found evidence for a set of genes with conserved intron content which was very highly enriched for genes involved in development, identified using DAVID (Table 2). Only the top 20 enriched terms are shown in Table 2. These include terms corresponding to the presence of homeobox DNA-binding domains, frequently involved in developmental gene regulation. To look for examples of conserved intron content among genes with higher intron content, we separated the human genes into four intron content size classes (1 – 5 kb, 5 – 20 kb, 20 – 50 kb and 50 – 100 kb), to account, to some extent, for the fact that patterns of intron length evolution may differ between genes with larger versus smaller introns. The proportion of genes from the other size classes (other than size class one, discussed above) that showed evidence of conserved intron content was smaller and we found no evidence for enrichment for the default DAVID gene sets (after multiplicity correction).

**Table 2:** **Gene set enrichment analysis** Gene set enrichment analysis of genes in size class 1 showing evidence of intron content conservation in mammals. Top twenty most significantly enriched terms are shown.

Category	Term	Count	***P*****-value**	FDR (BH)^1^
UP_SEQ_FEATURE	DNA-binding region:Homeobox	**20**	2 × 10^–10^	8 x 10^–8^
INTERPRO	Homeobox, conserved site	**20**	9 × 10^–10^	2 x 10^–7^
SP_PIR_KEYWORDS	Homeobox	**20**	2 × 10^–9^	4 x 10^–7^
INTERPRO	Homeobox	**19**	5 x 10^–9^	6 x 10^–7^
GOTERM_BP_FAT	Regulation of transcription, DNA-dependent	**41**	7 x 10^–9^	1 x 10^–5^
INTERPRO	Homeodomain-related	**19**	1 x 10^–8^	8 x 10^–7^
GOTERM_BP_FAT	Regulation of RNA metabolic process	**41**	1 x 10^–8^	8 x 10^–6^
GOTERM_BP_FAT	Positive regulation of transcription from RNA polymerase II promoter	**19**	1 x 10^–8^	6 x 10^–6^
GOTERM_BP_FAT	Positive regulation of biosynthetic process	**27**	3 x 10^–8^	1 x 10^–5^
GOTERM_BP_FAT	Positive regulation of cellular biosynthetic process	**27**	3 x 10^–8^	1 x 10^–5^
GOTERM_MF_FAT	Transcription regulator activity	**38**	7 x 10^–8^	2 x 10^–5^
GOTERM_BP_FAT	Positive regulation of gene expression	**24**	8 x 10^–8^	2 x 10^–5^
GOTERM_BP_FAT	Positive regulation of transcription, DNA-dependent	**21**	8 x 10^–8^	2 x 10^–5^
GOTERM_BP_FAT	Positive regulation of RNA metabolic process	**21**	8 x 10^–8^	2 x 10^–5^
GOTERM_BP_FAT	Positive regulation of macromolecule biosynthetic process	**25**	9 x 10^–8^	2 x 10^–5^
GOTERM_BP_FAT	Positive regulation of macromolecule metabolic process	**27**	1 x 10^–7^	2 x 10^–5^
GOTERM_BP_FAT	Positive regulation of nitrogen compound metabolic process	**25**	1 x 10^–7^	2 x 10^–5^
GOTERM_MF_FAT	Sequence-specific DNA binding	**25**	1 x 10^–7^	2 x 10^–5^
SMART	HOX	**19**	2 x 10^–7^	8 x 10^–6^
GOTERM_BP_FAT	Positive regulation of transcription	**23**	2 x 10^–7^	3 x 10^–5^

^1^Benjamini-Hochberg FDR corrected *P*-values

### Intronic insertions and deletions along lineages leading to human and mouse

To investigate evolution of intron length through insertion and deletion in greater detail we considered changes in intron length along the human and mouse lineages following divergence from their last common ancestor. Genomic sequences at ancestral nodes of the mammalian phylogeny were obtained from Ensembl. These sequences were inferred using a probabilistic method that has been shown to have high accuracy for the inference of insertion and deletion events. We mapped insertion and deletion events to introns.

**Figure 2:**
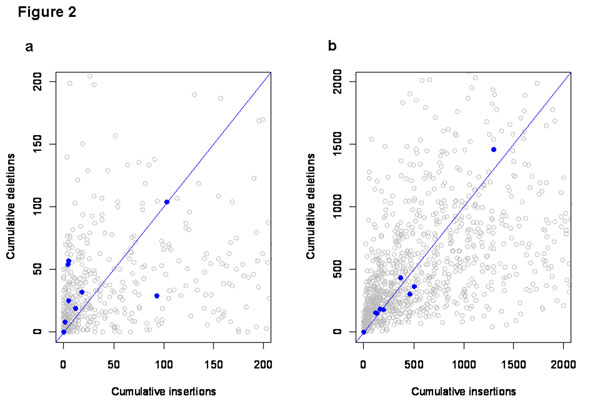
**Comparison of insertions and deletions** The sum of the insertions and deletions that have taken place in genes in size class 1 along the human (a) and mouse (b) lineages since divergence from their common ancestor. Blue points, corresponding to the genes that showed as much or more intron content conservation than *Hes7* are shown against a background of grey points, corresponding to all genes in the size class. All axes are truncated, but the truncated axes include all of the blue points. Grey points lying with cumulative insertions or deletions greater than **200** bp for human or **2000** bp for mouse are not shown.

Conservation of intron content could result from the absence of insertion and deletion events or from balance between insertion and deletion. For the 11 genes that showed as much conservation as *Hes7* across the mammalian panel, we found qualitative support for both effects. The total amount of insertion and deletion was lower in these genes but there were also some genes with substantial insertion and deletions in balance (Fig. 2). This was particularly evident in the case of indels along the lineage leading to mouse, however, as these genes were selected on the basis of their conservation in a panel that included humans and mouse this result should be considered to be illustrative only.

We also identified the complete set of genes in size class one that were annotated with a homeobox domain by Interpro [18]. To investigate whether the change in intron content since the common ancestor of human and mouse was less than expected, given the inferred numbers of indels we used a randomization procedure (*described in* Data and methods). We applied this procedure to the set of homeobox domain proteins and found that the change in intron content of these genes along both the human and mouse lineages was far less than expected, given the number of indels inferred to have occurred in each lineage and under the null assumption that the ratio of insertions and deletions and size distribution of these events did not differ for these genes compared to the rest of genes in the size class. In the case of human, the mean absolute change in intron content was 450 bp per gene, whereas the median of this value in the randomized data was 846 bp per gene. The randomized values were less than or equal to the observed value in 87 of 10,000 randomizations (one-tailed p = 0.009). In mouse the mean absolute change in intron length was just 9 bp per gene. The median of this quantity across randomizations was 398 bp per gene. The values in the random data were lower than or equal to the observed data in 33 of 10,000 randomizations (one-tailed p = 0.003).

### Contribution of conserved sequence elements to intron length conservation

**Figure 3:**
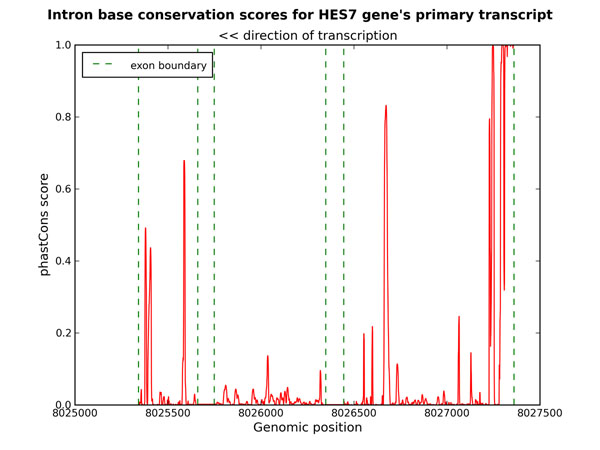
phastCons scores across introns of the Hes7 gene

From our results it appears that there is selection to prevent large changes in intron content in specific sets of genes, notably in genes involved in development and especially in early embryonic patterning. This selection could result from the presence of *cis* or *trans* (e.g. non-coding RNAs) regulatory elements within introns of these genes, which would be disrupted by insertions and even more so by deletions. However, the presence of regulatory elements would tend to increase the intron length [19], yet we found good evidence for classes of genes with well conserved intron content only among the genes with lower intron content. To test the contribution of conserved functional elements within intronic sequences on the conservation of intron content we obtained conservaton scores, calculated using phastCons, from the UCSC genome database. phastCons scores for the intronic regions of *Hes7* are shown as figure 3. While there is evidence of functional elements, these are relatively few and short and seem unlikely to prevent changes in intron length over evolutionary time. More quantitatively, we compared phastCons scores (averaged across all intronic positions) between the 144 genes with evidence of conserved intron content and the remainder of the genes in the smallest intron size class. The difference in mean phastCons scores was not significant (p = 0.33, Wilcoxon rank sum test). The difference in the proportion of conserved sites (phastCons scores > 0.95) was also non-significant (p = 0.69), suggesting that a greater proportion of conserved intronic functional elements is not the cause of the intron length conservation in these genes.

To explain our observations, conservation acting on functional elements would, perhaps, have to be balanced with selection for rapid induction, as rapidly induced genes have been shown to have short introns [20]. If the conservation of intron content is a consequence of balance between conservation of intronic functional elements and selection either for efficiency [21] or for rapid induction, this balance appears to be particularly significant for development-associated genes. For these genes, expression precision at the critical developmental junctures in which patterns are established in the developing embryo may be particularly crucial. An intriguing role for introns that has recently gained experimental support is in the establishment of oscillating patterns of gene expression through the control of time delays in expression [11]. These authors attributed a 19 minute delay to the presence of introns. However, the introns of *Hes7* are relatively short (< 2 kb) and RNA polymerase II processes nucleotides at a rate in the order of 2 kb per minute [22,23]. This may suggest either the existence of other functional elements within the intron that caused the delay in transcription or specific properties of the intron that result in an exceptionally slow transcription rate.

## Conclusion

Some theories of intron evolution have focussed on the energy cost associated with introns. Reduced intron lengths in highly expressed genes were proposed to result from selection for efficiency [21]. In support of this model, greater selective efficiency associated with larger effective population sizes of unicellular versus multicellular organisms, is associated with shorter introns [2,3]. Alternatively longer introns in highly and ubiquitously expressed genes may be associated with greater regulatory complexity and the preservation of regulatory elements in introns [19]. Selection to reduce energetic costs of transcription and for rapid induction of some genes as well as selection to preserve regulatory elements in highly regulated genes may all be important factors for intron evolution. However, comparison of genes with very conserved intron contents carried out here, suggests that there may be a substantial number of genes for which the size of the primary transcript is an important and conserved feature. These genes are enriched for key developmental processes, particularly the establishment of early embryonic patterns. Since time delays in gene induction have been shown to be important for some such genes, we propose that selection to conserve transcription time is an important factor in the evolution of intron lengths, particularly in development-associated genes. Our analysis involved a combination of a heuristic examination of intron content across a panel of mammalian species and a statistical randomization approach, which does not take into account all of the factors that may affect the fixation probabilities of insertions and deletions in introns. A better understanding of the evolution of intron sizes through insertion and deletion requires the development of evolutionary models of intron length evolution, though this is challenging because of the diversity of insertion and deletion events and the difficulty in modelling the constraints imposed by functional elements within the introns on the occurrence and size distribution of these events. If an appropriate model of neutral evolution by insertion and deletion can be derived, such a model could be used to identify and quantify purifying selection acting on intron lengths and, perhaps, to discover examples of positive selection acting on changes in intron length over a phylogeny or specific branches of a phylogenetic tree.

## Data and methods

Complete sets of gene models were downloaded from Ensembl release 62 [24] via BioMart for 19 mammalian species. These were *Homo sapiens*, *Bos taurus*, *Canis familiaris*, *Ornithorhynchus anatinus*, *Equus caballus*, *Erinaceus europaeus*, *Gorilla gorilla*, *Loxodonta africana*, *Monodelphis domestica*, *Myotis lucifugus*, *Microcebus murinus*, *Mus musculus*, *Oryctolagus cuniculus*, *Sus scrofa*, *Spermophilus tridecemlineatus*, *Tarsius syrichta*, *Tursiops truncatus*, *Vicugna pacos* and *Felis catus.* Species were selected to sample a broad range of mammalian evolutionary history. A phylogenetic tree of these taxa, obtained from the interactive tree of life [25,26] is provided for illustrative purposes (Fig. 1). For each Ensembl gene in each species we considered all annotated exons and calculated the total intronic content of the gene as the sum of the gaps between non-overlapping successive exons in the canonical transcript associated with the gene. Genes were divided into four classes, depending on the total intron content of the gene (1 – 5 kb, 5 – 20 kb, 20 – 50 kb and 50 – 100 kb). Orthologous groups of mammalian proteins were downloaded from OrthoDB [15]. We extracted the protein identifiers from OrthoDB corresponding to each of the mammalian species included in the study. Where more than one protein from a species was included in the same orthologous group, we selected one paralogue at random. For each orthologous group, with a representative in at least half of the mammalian species considered we determined the number of species in which the intron content, as defined above, was within 10% of the length of the intron content of the human gene. This was done separately for genes in different intron content classes. Functional analysis of genes with conserved intron content was carried out using DAVID [16,17]. For each size class, the genes for which the intron content showed evidence of conservation was used as the foreground set and the complete set of genes in the size class was used as the background set.

Genomic multiple sequence ancestor alignments, inferred using the Ensembl Enredo-Pecan-Ortheus pipeline [27], were downloaded from the comparative genomics section of the Ensembl FTP site. Insertions and deletions were inferred for ancestral sequences included in these alignments using a branch transducer method that has been shown to achieve high accuracy [28]. This allowed insertions and deletions to be placed on branches of the mammalian phylogeny. For reconstructed insertion and deletion events we focussed on the branches leading from the common ancestor of the euarchontoglires (which includes rodents and primates) to humans and mouse. All insertion and deletion events occurring within introns (at least 20 bp from exon-intron) boundaries were identified, based on Ensembl gene models. In this case, intronic indels were defined as indels that occur within the boundaries of the gene but not within 20 bp of any annotated exon associated with the gene.

### Randomization study of intron length evolution along the human lineage

For each intron size class we used the complete set of insertion and deletion events within the introns to define the null expectation of events in that intron size class. To test for evidence of purifying selection acting on the intron content of a gene or a set of genes we considered all of the insertion and deletion events in the gene(s) under consideration and for each event sampled an insertion or deletion from the total set of events in the introns of genes in that class. Given a set of insertions and deletions randomly sampled in this way we calculated the change in intron content implied by this set of insertions and deletions (sum of insertion lengths minus the sum of the lengths of the deletions), separately along the human and mouse lineages. This was repeated 10,000 times and in each case the implied change in intron content in the randomized data was compared to the change in intron content, given the actual insertions and deletions inferred to have occurred. The number of times the absolute value of the change in intron content in the randomized data was less than or equal to the absolute value of the change in intron content in the observed data was used to calculate a p-value, with a separate p-value calculated for the human and mouse lineages. These p-values indicate the probability of observing as little or less variation in the intron content of a gene or set of genes, given the number of insertion and deletion events that have occurred and under the assumption that these indel events have the same distribution as events in other genes in the same intron size class.

## Authors' contributions

CS conceived the project. CS and PK performed the analysis and co-wrote the manuscript.

## Competing interests

The authors declare that they have no competing interests.
